# Excess Healthcare Costs and Resource Utilisation of Lyme Borreliosis in Germany: A Propensity Score–Matched Cohort Study

**DOI:** 10.1111/zph.13180

**Published:** 2024-09-05

**Authors:** Gordon Brestrich, Joanna Diesing, Nils Kossack, James H. Stark, Andreas Pilz, Holly Yu, Jochen Suess

**Affiliations:** ^1^ Pfizer Pharma GmbH Berlin Germany; ^2^ Scientific Institute for Health Economics and Health System Research (WIG2 GmbH) Leipzig Germany; ^3^ Vaccines, Antivirals, and Evidence Generation, Pfizer Biopharma Group Cambridge Massachusetts USA; ^4^ Pfizer Corporation GmbH Vienna Austria; ^5^ Health Economics and Outcomes Research, Pfizer Biopharma Group Collegeville Pennsylvania USA; ^6^ Brehm Memorial Center (BREHM WORLD) Renthendorf Germany

**Keywords:** disseminated Lyme, Germany, healthcare cost, healthcare resource utilisation, Lyme borreliosis

## Abstract

**Aim:**

Lyme borreliosis (LB) is the most common tick‐borne disease in Germany; however, data on the economic burden of LB are limited. In this study, we aim to report healthcare costs, healthcare resource utilisation (HCRU) and diagnostic consumption associated with LB by clinical manifestation.

**Method:**

Using specific case definitions, patients with localised disease (erythema migrans [EM]) or disseminated disease (Lyme arthritis [LA], Lyme neuroborreliosis [LNB] and other rarer manifestations [OTH]) were identified from a claims database in 2016 and followed up for 3 years (2016–2019). After propensity score matching, excess costs and HCRU were calculated as the differences between each LB cohort and the matched control cohort.

**Results:**

On a per‐patient basis, the excess all‐cause healthcare cost was €130 for EM during Quarter 1 of Year 1, and €1539 for LA, €3248 for LNB and €4137 for OTH during Year 1. Only for OTH, additional €1860 was observed in Year 2. No increase in costs was observed in Year 3. When extrapolated to all German patients with statutory health insurance, LB was associated with €64.5 million in excess costs. Although disseminated manifestations only accounted for 7.8% of all LB cases, they were responsible for 66% of overall costs. In addition, LB patients consumed healthcare resources of 1.4 million excess outpatient visits, 13,000 excess hospitalisations, 96,000 ELISAs and 65,000 Western blots.

**Conclusion:**

This study shows the substantial economic burden of LB to the German healthcare system.

**Summary**

- Study assesses the first‐time healthcare costs and healthcare resource utilisation for individual clinical Lyme borreliosis manifestations across all healthcare settings in Germany.
- Previous economic evaluations are outdated and limited in interpretation because they used a sensitive but not specific case definition, focused on a specific sector of the healthcare system (inpatients or outpatients).
- Results can be used in health economic models to guide public health experts on the decision‐making to implement new Lyme borreliosis treatments and preventions.

## 1 Introduction

Lyme borreliosis (LB), caused by the spirochete *Borrelia burgdorferi* sensu lato (Bb), is the most common tick‐borne disease in Germany (Akmatov et al. 2021; Bohmer et al. 2021; Enkelmann et al. 2018). Human risk for LB is multifactorial and variably influenced by climate, land use and ecological conditions that support tick hosts and animal reservoirs for Bb, and individual behaviours that increase or decrease encounters with Bb‐infected ticks (Bouchard et al. 2019).

Due to climatic and environmental changes, leading to a longer tick activity period, the number of LB cases is expected to increase in Europe in the future (Lindgren, Talleklint, and Polfeldt 2000). LB can manifest in various forms—early symptoms can include a skin rash known as erythema migrans (EM) and flu‐like symptoms, while disseminated disease can lead to neurologic, musculoskeletal and other rarer manifestations (e.g., Lyme neuroborreliosis [LNB], Lyme arthritis [LA] and others [OTH]) (Schwartz et al. 2017). Most patients with LB will recover completely after antibiotic treatment; however, approximately 5%–10% of patients will experience persistent symptoms after treatment that may last months or years (Ursinus et al. 2021).

Previous studies using administrative healthcare claims data estimated the incidence of LB to be 261–429 outpatient cases/100,000 and nine hospitalisations/100,000 (Akmatov et al. 2021). However, only two studies have estimated the economic impact of LB on the German healthcare system. Both studies analysed a claims database containing a representative patient sample of statutory health‐insured persons. The yearly economic impact was extrapolated to all of Germany and was estimated to be €14.9 million for outpatient care for the years 2007–2008 and €23.7 million for inpatient care for the years 2007–2011 (Lohr et al. 2015; Muller et al. 2012). However, these studies used the ICD–10 GM diagnosis code A69.2 alone to identify LB cases without further treatment or diagnostic–related criteria, a method which may lead to overestimation of disease burden due to the use of a less specific algorithm. To overcome these limitations, this study applied a more specific case definition—an LB diagnosis code and an antibiotic prescription and/or diagnostic test order—to identify medically attended LB cases. These parameters plus additional manifestation diagnosis codes were used to define specific disease manifestations, which included EM, LA, LNB and a group of all other manifestations (OTH). Previous studies also focused on a specific sector of the healthcare system (inpatients or outpatients) and reported data from more than a decade ago (Lohr et al. 2015; Muller et al. 2012). As such, updated cost estimates are needed to provide a comprehensive, current understanding of the economic burden associated with LB in Germany to guide current and future prevention strategies and decision‐making.

To address the research gaps, we assessed the total healthcare costs, costs by sector (inpatient, outpatient and medication) and healthcare resource utilisation (HCRU) attributed to LB in Germany during the period 2016–2019. Using a specific case definition, patients with localised disease (i.e., EM) and disseminated disease (i.e., LA, LNB and other rarer manifestations [OTH]) were identified from a claims database; accordingly, all cost and HCRU analyses were stratified by clinical manifestation.

## 2 Materials and Methods

### 2.1 Data Source and Study Design

**FIGURE 1:**
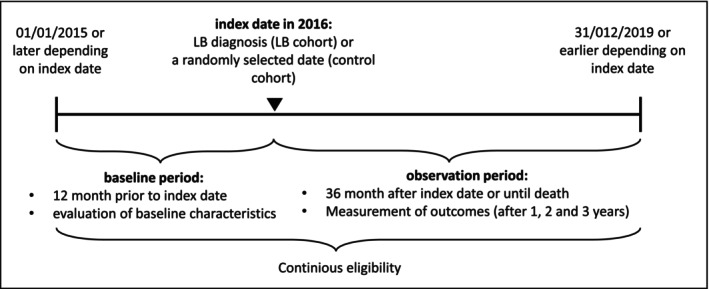
Study design.

This study used claims data from the WIG2 (Scientific Institute for Health Economics and Health System Research) database in Germany. The WIG2 database contains approximately 4.5 million patients under statutory health insurance (SHI) or 5% of the German SHI population (Stander et al. 2020). Healthcare claims data are provided to WIG2 solely for research purposes and are fully anonymised, therefore, no ethical approval was required. A retrospective, matched cohort study was conducted. LB patients were identified in the study year of 2016 and followed up from the index date for up to 3 years (also described as Year 1, Year 2 and Year 3 of the follow‐up period) (Figure 1). Patients were classified into one of the four LB cohorts (i.e., EM, LA, LNB and OTH) or a control cohort (i.e., without an LB diagnosis).

### 2.2 Study Population—The LB Cohort and the Control Cohort

For the LB cohorts, the index date was the date of the first recorded diagnosis of LB in 2016. For the control cohort, the index date was a date in 2016 that was matched to the index date of an LB case. For the LB cohorts and the control cohorts, patients of all ages were included in the analysis if they had: (Akmatov et al. 2021) continuous insurance from birth or at least four quarters prior to the quarter of the index date (baseline period) and (Bohmer et al. 2021) continuous insurance since the index date in 2016 until death or the end of the 3‐year follow‐up. For each LB cohort, patients were also required to have a manifestation‐specific diagnosis (i.e., EM, LA, LNB or OTH). Patients with EM were identified based on a main or secondary inpatient diagnosis code, or an outpatient diagnosis code (ICD‐10 GM code A69.2), plus a prescription of recommended antibiotic treatment for LB. For disseminated cases, patients were identified based on the main or secondary inpatient or outpatient diagnosis code of A69.2 plus a manifestation‐specific code (Table S1), an antibiotic prescription and a laboratory test order in the same quarter as the LB diagnosis.

### 2.3 Study Endpoints

Study endpoints measured during the follow‐up period (2016–2019) were all‐cause total healthcare costs, all‐cause inpatient costs, all‐cause outpatient costs and all‐cause medication costs. HCRU endpoints included the number of outpatient visits, LB‐specific diagnostics used (ELISAs and Western blots [WBs]) and the number of hospitalisations.

### 2.4 Propensity Score Matching

To adjust for potential confounders, propensity score matching (PSM) was conducted between each of the four LB cohorts (i.e., EM, LA, LNB and OTH) and a control cohort. For PSM, four baseline variables were selected. The risk for LB is highly associated with age, gender and region. Therefore, we included these parameters in the PSM. As we compared absolute healthcare costs between LB patients and a control population, we also matched for Charlson comorbidity index (CCI) as comorbid conditions can have a high impact on annual healthcare costs. The 1:4 PS matched cohorts (case:control) were created using the nearest‐neighbour methodology with a calliper of 0.01. The balance of baseline variables was checked based on standardised mean differences (SMD) before and after PSM. Variables with SMDs below 0.10 after matching were considered well balanced. Assessment of baseline differences was conducted for the covariates included in the propensity score model as well as for the baseline differences in HCRU and costs.

### 2.5 Statistical Analyses

Incidence of EM and disseminated LB (LA, LNB and OTH) were calculated by the proportion of patients with new onset of EM or disseminated LB (LA, LNB and OTH) within the total population and expressed as per 100,000 population per year. The matched study cohorts and controls were used to assess excess healthcare costs and HCRU during the follow‐up period. After balancing was achieved, excess healthcare costs and HCRU were estimated by the differences between each of the four LB cohorts and the matched control cohorts. A paired *t*‐test was performed to test if differences were statistically significant between each LB cohort and the matched control cohort quarterly or yearly during the follow‐up. As most EM cases can be successfully treated with 2–4 weeks of antibiotic therapy, the analyses of EM case costs focused on the short‐term quarterly costs in the 1st year after the index date. For disseminated LB manifestations, a longer follow‐up period (up to 3 years) was used to capture the potentially associated long‐term costs and HCRU. Healthcare costs and HCRU were reported as per patient per quarter (EM) or per patient per year (LA, LNB or OTH). Lastly, estimated excess healthcare cost and HCRU were extrapolated to the overall SHI population, using the manifestation‐specific incidence estimated in this study for 2016 and the total number of SHI members based on KM6 statistics (Bundesministerium für Gesundheit n.d.). Confidence intervals for excess costs were calculated using normal approximation and bootstrapping.

## 3 Results

### 3.1 Patient Characteristics Before PSM

A total of 10,690 EM patients (incidence 236.5/100,000 population per year), 324 LA patients (incidence 7.4/100,000 population per year), 422 LNB patients (incidence 9.7/100,000 population per year) and 132 OTH patients (incidence 2.8/100,000 population per year) were identified in the WIG2 database in 2016.

### 3.2 PS‐Matched Study Cohorts

A total of 2,826,463 control patients were identified and used for matching to the four LB cohorts. All LB patients were successfully matched. A total of 10,690 EM patients were matched to 42,760 controls, 324 LA patients to 1296 controls, 422 LNB patients to 1288 controls and 132 OTH patients to 528 controls (Table S2). The characteristics of each LB cohort and the matched control cohorts were well balanced, with the standardised mean difference of all matching variables below 0.10. All healthcare costs and HCRU variables were similar between the four LB cohorts and matched control cohorts during the baseline year with the exception of outpatient visits for LA and LNB where LB patients showed slightly more visits at baseline, which could reflect some additional healthcare utilisation before a proper LB diagnosis was made for disseminated cases.

### 3.3 Healthcare Costs Among Matched Cohorts

All‐cause total healthcare costs in the 3 years after the index date were compared between each LB cohort and the matched control cohort (Table 1). In Year 1, patients with EM had similar all‐cause total healthcare costs when compared with the matched control cohort; in contrast, patients with disseminated disease (LA, LNB or OTH) incurred significantly higher all‐cause total healthcare costs. For patients with OTH, the cost difference was also significant in Year 2. To determine whether there were significant cost differences within Year 1 for the EM cohort, costs were also analysed for each quarter in Year 1. Only during Quarter 1, patients with EM showed a significantly higher cost compared with the control cohort (Table 1).

**TABLE 1:** Comparison of total costs, outpatient visits and hospitalisations between Lyme borreliosis (LB) manifestation cohorts and control cohorts.

LB manifestation	Variable	Mean LB patient (95% CI)	Mean control (95% CI)	Difference of LB patient vs. control	*p*
EM	Total costs Y1	€2563 (2451–2674)	€2544 (2467–2620)	€19 (−111 to 150)	0.817
	Total costs Y2	€2481 (2367–2596)	€2667 (2594–2739)	−€185 (−310 to −61)	0.020
	Total costs Y3	€2597 (2483–2711)	€2701 (2625–2777)	−€105 (−234 to 25)	0.206
	Total costs Q1	€723 (688–758)	€593 (569–618)	€130 (88 to 171)	0.001
	Outpatients visits Y1	21.9 (21.6–22.2)	17.5 (17.3–17.7)	4.4 (4.1 to 4.7)	0.001
	Outpatients visits Y2	18.7 (18.4–19.0)	17.4 (17.2–17.5)	1.3 (1.0 to 1.7)	0.001
	Outpatients visits Y3	18.5 (18.5–18.8)	17.3 (17.1–17.5)	1.2 (0.9 to 1.6)	0.001
	Hospitalisations Y1	0.30 (0.28–0.31)	0.27 (0.26–0.28)	0.03 (0.01 to 0.04)	0.001
	Hospitalisations Y2	0.26 (0.25–0.28)	0.27 (0.26–0.28)	−0.01 (−0.02 to 0.00)	0.219
	Hospitalisations Y3	0.26 (0.25–0.28)	0.27 (0.26–0.28)	0.00 (−0.01 to 0.02)	0.652
LA	Total costs Y1	€4278 (3505–5051)	€2739 (2333–3145)	€1539 (820 to 2258)	0.001
	Total costs Y2	€3703 (1863–5543)	€2958 (2596–3320)	€745 (−192 to 1682)	0.213
	Total costs Y3	€2926 (2290–3562)	€3035 (2546–3524)	−€109 (−931 to 714)	0.836
	Outpatients visits Y1	28.7 (26.8–30.7)	15.9 (15.0–16.7)	12.9 (11.3 to 14.5)	0.001
	Outpatients visits Y2	19.6 (17.8–21.4)	16.1 (15.1–17.1)	3.6 (1.8 to 5.3)	0.001
	Outpatients visits Y3	20.3 (18.3–22.3)	16.8 (15.8–17.9)	3.5 (1.6 to 5.3)	0.003
	Hospitalisations Y1	0.61 (0.47–0.76)	0.30 (0.25–0.35)	0.32 (0.22 to 0.42)	0.001
	Hospitalisations Y2	0.27 (0.19–0.35)	0.29 (0.24–0.33)	−0.02 (−0.09 to 0.06)	0.719
	Hospitalisations Y3	0.30 (0.21–0.39)	0.27 (0.23–0.31)	0.03 (−0.05 to 0.10)	0.588
LNB	Total costs Y1	€6513 (5762–7265)	€3265 (2920–3610)	€3248 (2620 to 3877)	0.001
	Total costs Y2	€3632 (2973–4290)	€3537 (3168–3906)	€95 (−552 to 741)	0.818
	Total costs Y3	€3856 (3098–4614)	€3040 (2619–3460)	€816 (78 to 1554)	0.083
	Outpatients visits Y1	28.0 (26.2–29.7)	18.6 (17.6–19.6)	9.4 (7.6 to 11.2)	0.001
	Outpatients visits Y2	21.8 (20.1–23.4)	18.0 (17.0–19.0)	3.7 (2.0 to 5.5)	0.001
	Outpatients visits Y3	21.1 (19.5–22.7)	17.3 (16.4–18.3)	3.7 (2.1 to 5.4)	0.001
	Hospitalisations Y1	1.18 (1.05–1.31)	0.35 (0.30–0.39)	0.83 (0.75 to 0.92)	0.001
	Hospitalisations Y2	0.41 (0.31–0.50)	0.36 (0.31–0.41)	0.04 (−0.004 to 0.13)	0.428
	Hospitalisations Y3	0.40 (0.31–0.49)	0.32 (0.28–0.36)	0.08 (0.00 to 0.15)	0.119
OTH	Total costs Y1	€6557 (4250–8865)	€2421 (1710–3131)	€4137 (2673 to 5601)	0.001
	Total costs Y2	€5041 (2884–7197)	€3181 (2664–3697)	€1860 (665 to 3.055)	0.015
	Total costs Y3	€3469 (2293–4645)	€3461 (2815–4107)	€8 (−1128 to 1144)	0.991
	Outpatients visits Y1	29.6 (26.4–32.7)	20.0	9.6 (6.3 to 12.9)	0.001
	Outpatients visits Y2	22.9 (19.9–26.0)	20.6	2.4 (−0.9 to 5.7)	0.195
	Outpatients visits Y3	22.9 (19.8–25.9)	20.0	2.9 (−0.4 to 6.1)	0.119
	Hospitalisations Y1	0.80 (0.58–1.01)	0.28	0.51 (0.38 to 0.64)	0.001
	Hospitalisations Y2	0.48 (0.29–0.67)	0.41 (0.33–0.49)	0.07 (−0.08 to 0.22)	0.508
	Hospitalisations Y3	0.27 (0.13–0.41)	0.31 (0.25–0.38)	−0.04 (−0.16 to 0.08)	0.628

**FIGURE 2:**
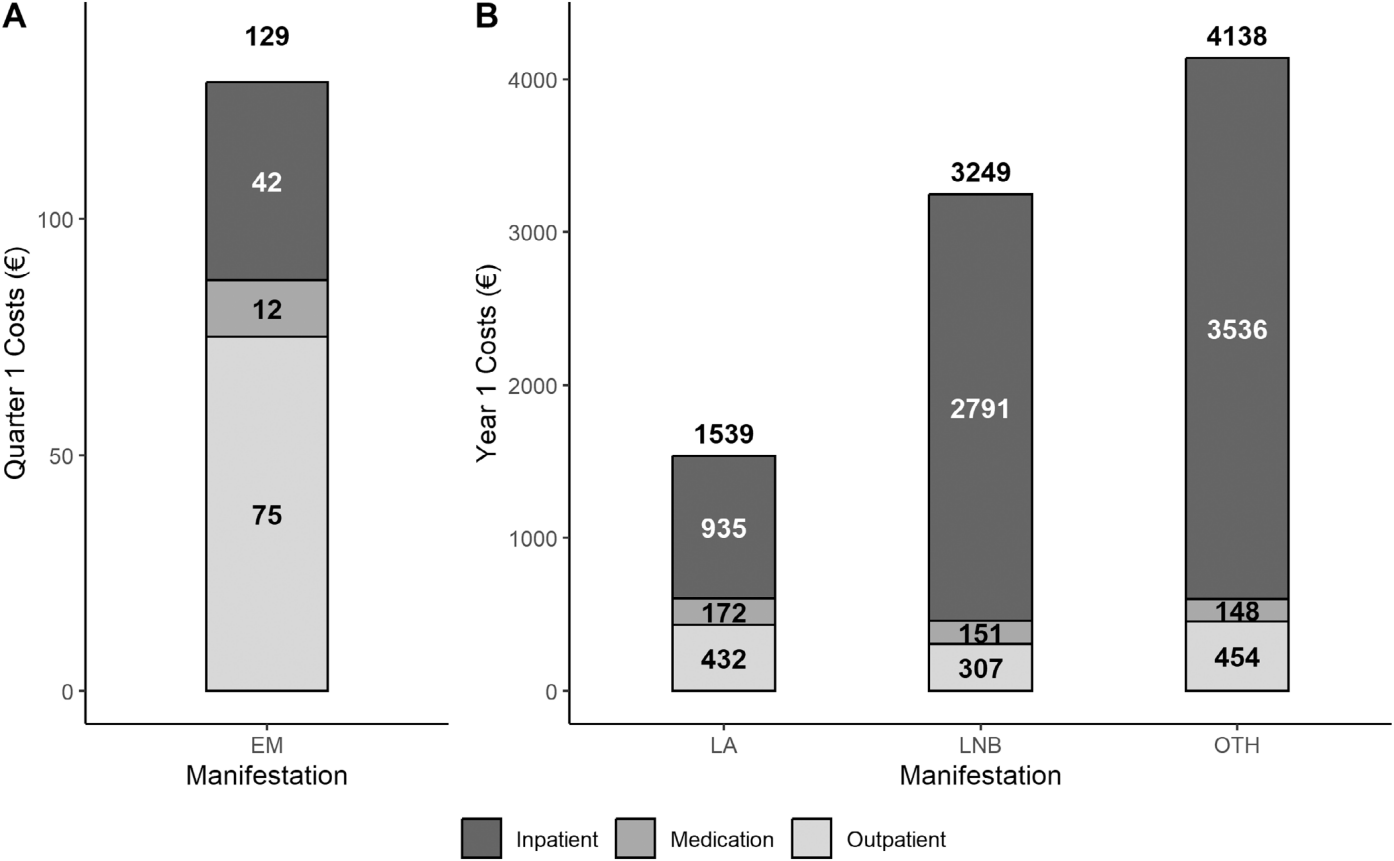
Excess all‐cause healthcare costs per patient by Lyme borreliosis (LB) manifestation and healthcare sector. (A) Excess inpatient, outpatient and medication costs of EM in Quarter 1 of Year 1 after LB diagnosis. (B) Excess inpatient, outpatient and medication costs of disseminated manifestations in Year 1 after LB diagnosis.

Figure 2 details the all‐cause healthcare costs attributed to LB by manifestation and by healthcare sector. Compared with control patients, patients with EM incurred an additional €130 (95% CI: 88–171) in Quarter 1 of Year 1, most of which was due to outpatient care (€75). In patients with disseminated manifestations (LA, LNB or OTH), the additional costs attributed to LB in Year 1 ranged from €1539 (95% CI: 820–2258) for LA to €3248 (95% CI: 2620–3877) for LNB and €4137 (95% CI: 2673–5601) for OTH. In Year 2, an additional €1860 was observed in patients with OTH manifestations. For disseminated LB, costs were primarily driven by inpatient costs (Figure 1). Of the three cost categories (inpatient, outpatient and medication), medication costs were the lowest across all four manifestations (Figure 2).

### 3.4 HCRU Among Matched Cohorts

**FIGURE 3:**
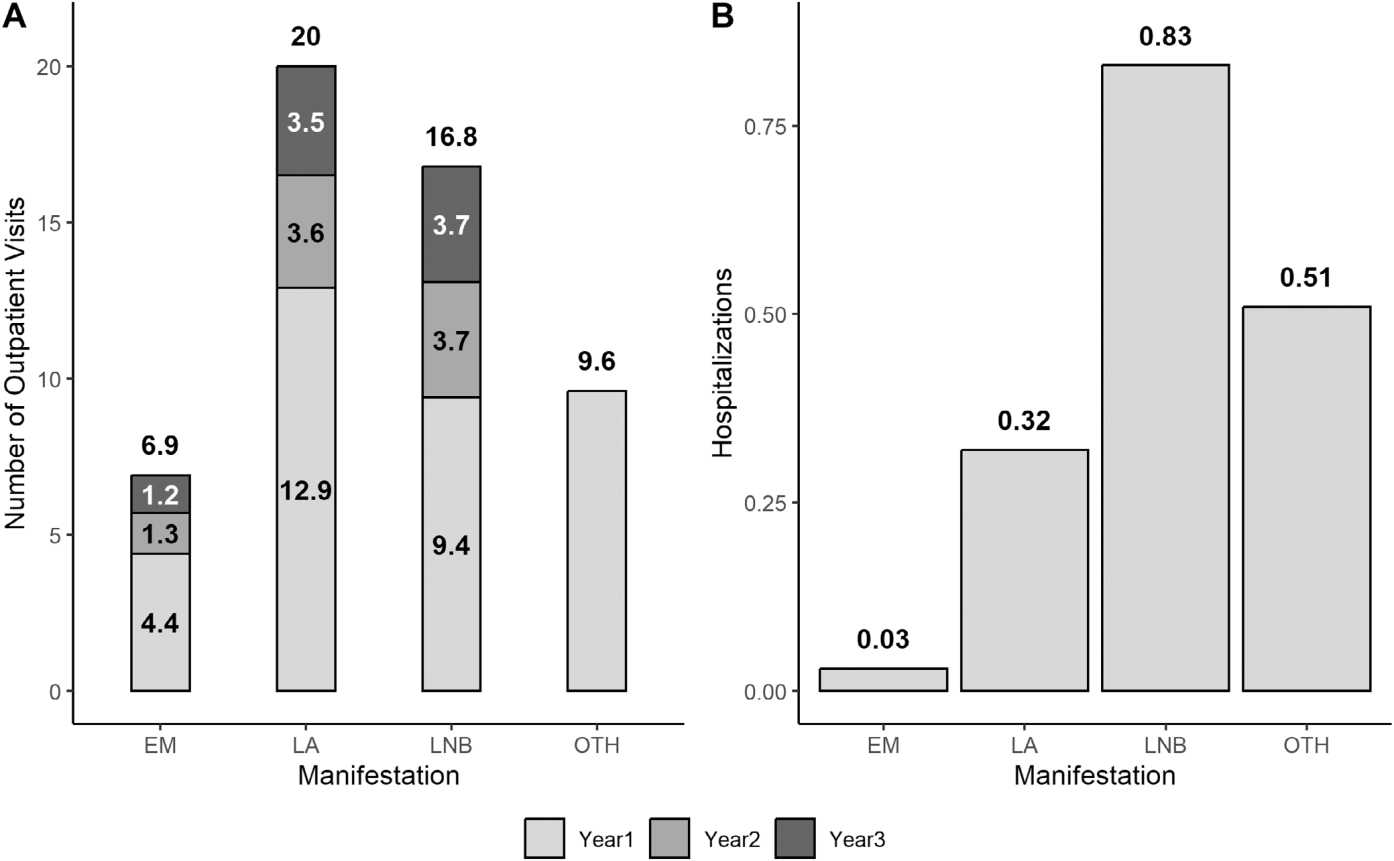
Number of excess outpatient visits and excess hospitalisations per patient by Lyme borreliosis (LB) manifestation and study year. (A) Excess outpatient visits in Year 1, Year 2 and Year 3 after LB diagnosis. (B) Excess hospitalisations in Year 1 after LB diagnosis.

Compared with matched control cohorts, use of outpatient care was significantly higher in patients with EM, LA or LNB for each year of the 3‐year follow‐up period (Table 1). For patients with OTH, only the difference in Year 1 was significant. The number of excess outpatient visits was highest in Year 1 for all manifestations. Over the 3‐year follow‐up, the excess number of outpatient visits ranged from 7 (EM) to 19.9 (LA) per case (Figure 3). Similarly, a significantly higher number of excess hospitalisations was observed for all LB manifestations in Year 1. Differences were not significant in Year 2 or Year 3. LNB was associated with the highest number of excess hospitalisations (0.83 excess hospitalisations per case), while hospitalisations were lowest in the EM cohort (0.03 excess hospitalisations per case) (Figure 3). LB laboratory testing was analysed in the four LB cohorts (Table S3). Tests such as PCR and cell culture were minimally used (data not shown). In 2016, ELISA was used in 51% of EM cases and 47%–80% of disseminated cases; WB was used in 35% of EM cases and 35%–61% of disseminated cases.

### 3.5 Economic Impact

The economic burden was extrapolated to all SHI patients (86% of the overall German population) with an LB infection in 2016, considering the costs that were significantly higher (i.e., *p* value < 0.05) in LB patients than those in the control cohorts as shown in Table 1. LB in all SHI patients in Germany was associated with €64.5 (95% CI: 53.5–75.6) million excess costs with €60.8 (95% CI: 43.7–78.9) million occurring in Year 1 (only Quarter 1 of Year 1 for EM patients) and €3.7 (95% CI: 1.3–6.1) million in Year 2 (only for OTH patients). In addition, SHI patients with LB infection in 2016 consumed 1.4 million excess outpatient visits, 13,000 excess hospitalisations, 96,000 ELISAs and 65,000 WBs (Table 2). Detailed calculations to extrapolate costs are displayed in Table S3.

**TABLE 2:** Extrapolated healthcare costs and resource utilisation among all statutory health insurance (SHI) patients with Lyme borreliosis (LB) diagnosis in 2016.

Excess healthcare costs (95% CI) (€)	Excess costs associated with disseminated disease (95% CI) (€)	Number of excess outpatient visits (95% CI)	Number of excess hospitalisations (95% CI)	Number of ELISAs (95% CI)	Number of Western blots (95% CI)
64,553,073 (53,528,656–75,577,490)^a^	42,647,382^a^ (40,354,746–44,940,017)	1,421,610 (1,227,392–1,615,828)	13,099 (10,430–15,768)	95,853 (94,197–97,510)	65,307 (63,717–66,896)

^a^ Costs contain €3,721,308 that are caused by other rarer manifestation (OTH) manifestations in the second year after LB diagnosis.

## 4 Discussion

This large, retrospective study evaluated excess healthcare costs and HCRU among German patients with LB from 2016 to 2019. After controlling for differences in patient characteristics, this study demonstrated that LB patients incurred a substantial amount of excess healthcare costs and HCRU in comparison with matched control patients.

The healthcare costs for LB in Germany have been investigated in two studies (Lohr et al. 2015; Muller et al. 2012). Both studies employed a broad case definition using the general ICD‐10 diagnosis code A69.2 alone. In contrast, our study provided recent data for the years 2016–2019 and assessed inpatient, outpatient and medication costs from cohorts of LB patients using a specific case definition, which included ICD‐10 code A69.2 plus an antibiotic prescription for EM cases, and additionally, manifestation‐specific ICD‐10 codes and a laboratory test order for disseminated disease. Specific case definitions used in the current study avoid an overestimation of LB cases and thus provide more precise estimates of attributable LB economic burden on the healthcare system (Cocoros et al. 2023). Evidence that ICD‐10 codes alone can overestimate the LB burden includes the relatively low percentage of LB patients (62.5%) receiving an antibiotic treatment in the Müller study (Muller et al. 2012).

The excess all‐cause healthcare costs attributed to LB varied by manifestation. On a per‐patient basis, the excess all‐cause healthcare cost was €130 for EM during Quarter 1 of Year 1. This is expected as most EM cases resolve after a 10‐ to 14‐day course of antibiotics in the outpatient setting (German Medical Science 2017). Costs for EM were comparable to those estimated in Belgium and the Netherlands with €193 and €122, respectively (Geebelen, Devleesschauwer, et al. 2022; van den Wijngaard et al. 2017). More than 50% of the overall EM costs (€75 of €130) were generated in the outpatient setting, whereas hospitalisations due to EM were rare with 0.03 excess hospitalisations per EM patient in Year 1. This is consistent with other studies reporting an EM hospitalisation rate of 1.7%–2.0% (Enkelmann et al. 2018; Wilking and Stark 2014). Nonetheless, approximately one‐third of the overall costs (€42 of €130) in EM patients were due to inpatient costs, indicating that although rare, these hospitalisations are costly.

Compared to EM, patients with disseminated disease incurred considerably higher costs over a longer period of time. This is also expected, as disseminated LB can be difficult to diagnose and treat (Gaubitz et al. 2014; Rauer et al. 2020). For disseminated disease, excess costs per patient were €1539 for LA, €3248 for LNB and €4137 for OTH in Year 1 after diagnosis. For OTH, additional costs of €1860 occurred in Year 2. Although the long‐lasting burden of post‐treatment Lyme Disease (PTLD) on patients and the healthcare system is described, we could not detect additional costs in year 3 after LB infection. The reason may be that PTLD occurs only in 5%–10% of LB patients and patients can recover with time (Ursinus et al. 2021). These few patients in the database may not be enough to add significant cost to the overall LB population. A patient survey with long‐term follow‐up may be better a tool to measure these costs (Geebelen, Lernout, et al. 2022). These results are consistent with studies conducted in other EU countries: for disseminated LB, a Belgium study estimated a per‐patient cost of €5148 and a study in the Netherlands reported a per‐patient cost of €5700 (Geebelen, Devleesschauwer, et al. 2022; van den Wijngaard et al. 2017). For all disseminated manifestations, the primary cost driver was inpatient costs (>60% for LA and >85% for OTH). LNB was associated with the highest excess hospitalisation rate (0.83/patient), while LA was the lowest (0.32/patient); both values are consistent with prior German studies (Enkelmann et al. 2018; Wilking and Stark 2014). The highest number of outpatient visits was observed in patients with LA, followed by those with LNB. Compared with the control cohort, outpatient visits were significantly increased for LA and LNB up to 3 years after diagnosis, highlighting the long‐term impact of the disease.

Currently, German clinical guidelines recommend laboratory testing only for disseminated disease, while EM cases are clinically diagnosed only. Additionally, testing should follow a two‐tier process, with an ELISA first, then a confirmatory WB (German Medical Science 2017; Gaubitz et al. 2014; Rauer et al. 2020). However, our data showed that 51% of EM cases were tested with ELISA and 35% with WBs, indicating excessive use of LB diagnostics that can pose an additional burden to the healthcare system. Similar results on excessive LB testing have been reported in other European countries (Esposito et al. 2013; Petrulioniene et al. 2021; Vanthomme et al. 2012). Only 47% of LNB and 77% of LA cases were tested with ELISA. These findings indicate low adherence of healthcare professionals to clinical guidelines.

The extrapolation of the cost, HCRU and diagnostic consumption to all SHI patients in Germany revealed substantial excess healthcare costs (€64.5 million) attributable to LB, including inpatient, outpatient and medication costs. Other German claims studies estimated lower costs (€23.7 million inpatient and €14.9 million outpatient costs; Lohr et al. 2015; Muller et al. 2012). The outpatient study by Müller et al. (2012) only considered laboratory testing and antibiotic treatments without including other outpatient costs such as consultation costs; therefore, the reported costs only represented a fraction of the total outpatient costs. On the other hand, Müller et al. (2012) included privately insured patients who may have higher healthcare costs than SHI patients. In the study by Lohr et al. (2015) only inpatients with a primary LB diagnosis were included; as patients with a secondary diagnosis were not considered, this may be an underestimation of inpatient costs. Our study comprehensively assessed cases identified using both primary and secondary diagnoses and analysed costs stratified by LB manifestation in Germany. Although disseminated manifestations only accounted for 7.8% of all LB cases in 2016, they were responsible for 66% of the overall excess costs (€42.6 million of €64.5 million). This is well in line with studies from Belgium and the Netherlands where disseminated manifestations caused 62% and 51%, respectively (Geebelen, Devleesschauwer, et al. 2022; van den Wijngaard et al. 2017).

This study has limitations. First, as an inherent limitation to observational studies, even with PSM to balance patient characteristics, residual confounding due to unmeasured variables may still exist. Especially, LNB and LA cohorts had slightly more outpatient visits at baseline after PSM compared to controls, which might reflect some additional healthcare utilisation leading up to a delayed LB diagnosis. Without further evidence, we cannot determine whether our approach would lead to an overestimation of costs, due to cases being sicker at baseline than controls or to underestimation of costs, by accounting only for costs incurred after LB diagnosis. Second, the claims database did not contain information on occupation or outdoor activity and these variables could not be used in PSM. Both variables are known to increase the risk for LB. Therefore, the identified LB population may spend more time outdoors than the controls. This may have led to an underestimation of cost as people spending more time outdoors tend to be healthier (Lear et al. 2017). Third, although we applied a specific case definition to identify LB patients, there may be coding inaccuracies leading to disease misclassification. Fourth, although we comprehensively estimated direct healthcare costs by disease manifestation, indirect costs were not included in the analysis and thus were not incorporated in the estimated economic burden of LB. Lohr et al. estimated €7.1 million indirect costs due to productivity loss which accounted for 23% of total costs in their study (Lohr et al. 2015). Lastly, results from this study may not be generalisable to the population at large in Germany, given that the study was focused on patients under SHI and privately insured patients were not included in the analysis.

In this large, contemporary analysis using specific case definitions for all clinical LB manifestations, patients with LB incurred substantially higher healthcare costs and HCRU compared with matched control patients without LB. The yearly economic burden of LB on the German healthcare system was substantial and was primarily driven by the costs associated with disseminated disease. Although patients with EM had a lower cost on a per‐patient basis, the large number of cases summed to substantial expenditures.

## Author Contributions

Conceptualisation, G.B., J.D., J.H.S., A.P. and J.S.; methodology, G.B., J.D., H.Y. and J.H.S.; software and formal analysis, N.K.; investigation and data curation, G.B. and J.D.; resources, supervision, and funding acquisition, J.H.S.; writing – original draft preparation and visualisation, G.B.; writing – review and editing, G.B., J.D., N.K., J.H.S., H.Y., A.P. and J.S.; All authors have read and agreed to the published version of the manuscript.

## Conflicts of Interest

G.B., J.H.S., A.P. and H.Y. are employees of Pfizer in Germany, Austria and the United States and were involved in the design of this study, developing the study protocol and analysing the data. J.D. and N.K. are employees of WIG2 GmbH, which is an independent institute and paid consultant to Pfizer Pharma GmbH for designing the study, carrying out the analysis, interpreting the results and writing the manuscript. J.S. received an honorarium from Pfizer Pharma GmbH in connection with designing the study and interpreting the results. J.S. received no further funding from another company or other partners.

## Supporting information


**Table S1.** List of ICD‐10 GM codes to identify EM and disseminated LB.

**Table S2.** Matching parameters and results.

**Table S3.** Detailed calculations to extrapolate costs.

## Data Availability

The datasets generated and analysed during the current study are not publicly available due to data protection laws. Raw dataset data are not publicly available to preserve individuals' privacy under the European General Data Protection Regulation.

## References

[zph13180-bib-0001] Akmatov, H. J. , L. Dammertz , C. Kohring , J. Heuer , and J. Bätzing . 2021. “Bundesweite und Kleinräumige Kennzahlen zur Morbidität von Lyme‐Borreliose in Deutschland Anhand Vertragsärztlicher Abrechnungsdaten, 2010 bis 2019.” Zentralinstitut für die Kassenärztliche Versorgung in Deutschland (Zi) 219: 1–9. 10.20364/VA-21.06.

[zph13180-bib-0002] Bohmer, M. M. , K. Ens , S. Bohm , S. Heinzinger , and V. Fingerle . 2021. “Epidemiological Surveillance of Lyme Borreliosis in Bavaria, Germany, 2013–2020.” Microorganisms 9, no. 9: 1872. 10.3390/microorganisms9091872. 34576768 PMC8467410

[zph13180-bib-0003] Bouchard, C. , A. Dibernardo , J. Koffi , H. Wood , P. A. Leighton , and L. R. Lindsay . 2019. “N Increased Risk of Tick‐Borne Diseases With Climate and Environmental Changes.” Canada Communicable Disease Report 45, no. 4: 83–89. 10.14745/ccdr.v45i04a02. 31285697 PMC6587693

[zph13180-bib-0004] Bundesministerium für Gesundheit . “GKV‐Members and Jointly Insured Family Members on July 1st.” https://www.gbe‐bund.de/gbe/pkg_olap_tables.prc_set_hierlevel?p_uid=gast&p_aid=95846962&p_sprache=D&p_help=2&p_indnr=249&p_ansnr=91109527&p_version=2&p_dim=D.000&p_dw=3737&p_direction=drill.

[zph13180-bib-0005] Cocoros, N. M. , S. A. Kluberg , S. J. Willis , et al. 2023. “Validation of Claims‐Based Algorithm for Lyme Disease, Massachusetts, USA.” Emerging Infectious Diseases 29, no. 9: 1772–1779. 10.3201/eid2909.221931. 37610117 PMC10461665

[zph13180-bib-0006] Enkelmann, J. , M. Bohmer , V. Fingerle , et al. 2018. “Incidence of Notified Lyme Borreliosis in Germany, 2013–2017.” Scientific Reports 8, no. 1: 14976. 10.1038/s41598-018-33136-0. 30297731 PMC6175818

[zph13180-bib-0007] Esposito, S. , E. Baggi , A. Villani , et al. 2013. “Management of Paediatric Lyme Disease in Non‐Endemic and Endemic Areas: Data From the Registry of the Italian Society for Pediatric Infectious Diseases.” European Journal of Clinical Microbiology & Infectious Diseases 32, no. 4: 523–529. 10.1007/s10096-012-1768-6. 23109197

[zph13180-bib-0008] Gaubitz, M. , F. Dressler , H. I. Huppertz , A. Krause , and Kommission Pharmakotherapie . 2014. “Diagnosis and Treatment of Lyme Arthritis. Recommendations of the Pharmacotherapy Commission of the Deutsche Gesellschaft fur Rheumatologie (German Society for Rheumatology).” Zeitschrift für Rheumatologie 73, no. 5: 469–474. 10.1007/s00393-014-1370-7. 24924733

[zph13180-bib-0009] Geebelen, L. , B. Devleesschauwer , T. Lernout , et al. 2022. “Lyme Borreliosis in Belgium: A Cost‐of‐Illness Analysis.” BMC Public Health 22, no. 1: 2194. 10.1186/s12889-022-14380-6. 36443755 PMC9703731

[zph13180-bib-0010] Geebelen, L. , T. Lernout , B. Devleesschauwer , et al. 2022. “Non‐Specific Symptoms and Post‐Treatment Lyme Disease Syndrome in Patients With Lyme Borreliosis: A Prospective Cohort Study in Belgium (2016–2020).” BMC Infectious Diseases 22, no. 1: 756. 10.1186/s12879-022-07686-8. 36171561 PMC9518937

[zph13180-bib-0011] German Medical Science . 2017. Vol. 15, ISSN 1612‐3174. 10.3205/000255.

[zph13180-bib-0012] Lear, S. A. , W. Hu , S. Rangarajan , et al. 2017. “The Effect of Physical Activity on Mortality and Cardiovascular Disease in 130,000 People From 17 High‐Income, Middle‐Income, and Low‐Income Countries: The PURE Study.” Lancet 390, no. 10113: 2643–2654. 10.1016/S0140-6736(17)31634-3. 28943267

[zph13180-bib-0013] Lindgren, E. , L. Talleklint , and T. Polfeldt . 2000. “Impact of Climatic Change on the Northern Latitude Limit and Population Density of the Disease‐Transmitting European Tick *Ixodes ricinus* .” Environmental Health Perspectives 108, no. 2: 119–123. 10.1289/ehp.00108119. 10656851 PMC1637900

[zph13180-bib-0014] Lohr, B. , I. Muller , M. Mai , D. E. Norris , O. Schoffski , and K. P. Hunfeld . 2015. “Epidemiology and Cost of Hospital Care for Lyme Borreliosis in Germany: Lessons From a Health Care Utilization Database Analysis.” Ticks Tick Borne Disease 6, no. 1: 56–62. 10.1016/j.ttbdis.2014.09.004. 25448420

[zph13180-bib-0015] Muller, I. , M. H. Freitag , G. Poggensee , et al. 2012. “Evaluating Frequency, Diagnostic Quality, and Cost of Lyme Borreliosis Testing in Germany: A Retrospective Model Analysis.” Clinical & Developmental Immunology 2012: 595427. 10.1155/2012/595427. 22242037 PMC3254124

[zph13180-bib-0016] Petrulioniene, A. , D. Radzisauskiene , A. Paulauskas , and A. Venalis . 2021. “Lyme Disease Among Patients at an Ambulatory Unit in a Highly Endemic Country: Lithuania.” Medicina 57, no. 2: 184. 10.3390/medicina57020184. 33669940 PMC7924869

[zph13180-bib-0017] Rauer, S. , S. Kastenbauer , H. Hofmann , et al. 2020. “Guidelines for Diagnosis and Treatment in Neurology–Lyme Neuroborreliosis.” GMS German Medical Science 18: Doc03. 10.3205/000279. 32341686 PMC7174852

[zph13180-bib-0018] Schwartz, A. M. , A. F. Hinckley , P. S. Mead , S. A. Hook , and K. J. Kugeler . 2017. “Surveillance for Lyme Disease – United States, 2008–2015.” MMWR Surveillance Summaries 66, no. 22: 1–12. 10.15585/mmwr.ss6622a1. PMC5829628 29120995

[zph13180-bib-0019] Stander, S. , M. Ketz , N. Kossack , et al. 2020. “Epidemiology of Prurigo Nodularis Compared With Psoriasis in Germany: A Claims Database Analysis.” Acta Dermato‐Venereologica 100, no. 18: adv00309. 10.2340/00015555-3655. 33021323 PMC9309863

[zph13180-bib-0020] Ursinus, J. , H. D. Vrijmoeth , M. G. Harms , et al. 2021. “Prevalence of Persistent Symptoms After Treatment for Lyme Borreliosis: A Prospective Observational Cohort Study.” Lancet Regional Health Europe 6: 100142. 10.1016/j.lanepe.2021.100142. 34557833 PMC8454881

[zph13180-bib-0021] van den Wijngaard, C. C. , A. Hofhuis , A. Wong , et al. 2017. “The Cost of Lyme Borreliosis.” European Journal of Public Health 27, no. 3: 538–547. 10.1093/eurpub/ckw269. 28444236

[zph13180-bib-0022] Vanthomme, K. , N. Bossuyt , N. Boffin , and V. Van Casteren . 2012. “Incidence and Management of Presumption of Lyme Borreliosis in Belgium: Recent Data From the Sentinel Network of General Practitioners.” European Journal of Clinical Microbiology & Infectious Diseases 31, no. 9: 2385–2390. 10.1007/s10096-012-1580-3. 22391757

[zph13180-bib-0023] Wilking, H. , and K. Stark . 2014. “Trends in Surveillance Data of Human Lyme Borreliosis From Six Federal States in Eastern Germany, 2009–2012.” Ticks Tick Borne Disease 5, no. 3: 219–224. 10.1016/j.ttbdis.2013.10.010. 24656810

