# Supplementary material for: Excess Healthcare Costs and Resource Utilisation of Lyme Borreliosis in Germany: A Propensity Score–Matched Cohort Study
Source: Zoonoses Public Health. 2024 Sep 5;72(1):23–31. doi: 10.1111/zph.13180 (PMC11695699; doi:10.1111/zph.13180)
Supplement: Supplementary file 1 — Table S1. List of ICD‐10 GM codes to identify EM and disseminated LB. [file ZPH-72-23-s002.docx]

**Supplemental Table S1: List of ICD-10 GM codes to identify EM and disseminated LB.**

| **LB/EM Diagnosis** | **ICD-10 code** |
| --- | --- |
| Lyme disease/erythema chronicum migrans due to Borrelia burdorferei | A69.2 |
|  |  |
| **Diagnoses that may be related to LA** |  |
| **Diseases of the musculoskeletal system and connective tissue (only in combination with A69.2)** | **ICD-10 code** |
| Arthritis and polyarthritis due to other specified bacterial agents | M00.8–9 |
| Direct infections of joint in infectious and parasitic diseases classified elsewherea | M01.8 |
| Other arthritis | M13.0–9 |
| Arthropathies in other diseases classified elsewhere | M14.8 |
|  |  |
| **Diagnoses that may be related to LNB**  **Diseases of the nervous system code concerned**  **(only in combination with A69.2)** | **ICD-10 code** |
| Meningitis | G00.9, G01, G02, G03.0, G03.1, G03.8, G03.9 |
| Encephalitis, myelitis and encephalomyelitis | G04.2, G04.8, G04.9, G05.0, G05.2, G05.8 |
| Bacterial meningoencephalitis and meningomyelitis, not elsewhere classified | G04.2 |
| Other encephalitis, myelitis and encephalomyelitis (Postinfectious encephalitis and encephalomyelitis NOS) | G04.8 |
| Disorders of trigeminal nerve | G50.8, G50.9 |
| Facial nerve disorders | G51.0, G51.8, G51.9 |
| Bell palsy (facial palsy) | G51.0 |
| Other disorders of facial nerve | G51.8 |
| Disorders of facial nerve, unspecified | G51.9 |
| Disorders of other cranial nerves | G51.0, G51.8, G51.9 |
| Cranial nerve disorders in diseases classified elsewhere | G53.1, G53.8 |
| Nerve root and plexus disorders | G54.0–5, G54.8–9 |
| Other polyneuropathies | G62.8–9 |
| Polyneuropathy in diseases classified elsewhere | G63.0 |
| Other disorders of peripheral nervous system | G64 |
|  |  |
| **Diagnoses that may be related to OTH** |  |
| **Diseases of the eye and adnexa**  **(only in combination with A69.2)** | **ICD-10 code** |
| Iridocyclitis | H20.0–1, H20.8–9 |
| Other disorders of iris and ciliary body | H21.8–9 |
| Disorders of iris and ciliary body in diseases classified elsewhere | H22.0, H22.1, H22.8 |
| Chorioretinal inflammation | H30.0–9 |
| Other disorders of choroid | H31.8–9 |
| Chorioretinal disorders in diseases classified elsewhere | H32.0, H32.08, H32.8 |
| **Diseases of the circulatory system**  **(only in combination with A69.2)** | **ICD-10 code** |
| Acute pericarditis | I30.0–9 |
| Pericarditis in diseases classified elsewhere | I32.1–8 |
| Acute myocarditis | I40.0–9 |
| Myocarditis in diseases classified elsewhere | I41.0, I41.2, I41.8 |
| Cardiomyopathy | I42.9 |
| Cardiomyopathy in diseases classified elsewhere | I43.0 |
| Atrioventricular and left bundle-branch block | I44.0–7 |
| Other conduction disorders | I45.0–5, I45.8–9 |
| Other heart disorders in diseases classified elsewhere | I52.0–8 |
| **Other specified disorders of the skin and subcutaneous tissue (only in combination with A69.2)** | **ICD-10 code** |
| Lymphocytoma, benign cutis | L98.8 |
